# Speciation-dependent intracellular reduction and nanostructure formation of selenium and tellurium in unicellular algae

**DOI:** 10.1093/mtomcs/mfag014

**Published:** 2026-05-04

**Authors:** Kohei Odaka, Kensuke Inaba, Yugo Takeuchi, Keita Hiraka, Daisuke Ogawa, Hirokazu Sugimori, Kazuhiro Kumagai, Akiko Hokura

**Affiliations:** Graduate School of Engineering, Tokyo Denki University, 5 Senju-Asahicho, Adachi, Tokyo 120-8551, Japan; Graduate School of Engineering, Tokyo Denki University, 5 Senju-Asahicho, Adachi, Tokyo 120-8551, Japan; NISSAN ARC, LTD., 1 Natsushima-cho, Yokosuka, Kanagawa 237-0061, Japan; Department of Applied Chemistry, School of Engineering, Tokyo Denki University, 5 Senju-Asahicho, Adachi, Tokyo 120-8551, Japan; NISSAN ARC, LTD., 1 Natsushima-cho, Yokosuka, Kanagawa 237-0061, Japan; Tokyo Metropolitan Industrial Technology Research Institute, 2-4-10, Aomi, Koto, Tokyo 135-0064, Japan; Tokyo Metropolitan Industrial Technology Research Institute, 2-4-10, Aomi, Koto, Tokyo 135-0064, Japan; Nanodimensional Standards Group, Research Institute for Material and Chemical Measurement National Metrology Institute of Japan (NMIJ), National Institute of Advanced Industrial Science and Technology (AIST), Tsukuba Central 5, 1-1-1 Higashi Tsukuba, Ibaraki 305-8565; Department of Applied Chemistry, School of Engineering, Tokyo Denki University, 5 Senju-Asahicho, Adachi, Tokyo 120-8551, Japan

## Abstract

Understanding how chalcogen elements taken up by biological systems change their chemical speciation is essential for elucidating their intracellular behaviour. However, knowledge of the uptake and transformation of selenium and tellurium oxyanions in unicellular algae remains limited. In this study, selenium and tellurium oxyanions in different oxidation states (VI or IV) were added to two unicellular algae, *Chlamydomonas reinhardtii* and *Pseudococcomyxa simplex*, and their intracellular accumulation and chemical speciation were systematically investigated. X-ray absorption fine structure analysis revealed that both selenium and tellurium underwent intracellular reduction irrespective of their initial oxidation states. However, the extent of reduction, accumulation efficiency, and final chemical speciation differed markedly depending on both the oxidation state of the added oxyanion and the algal species, indicating shared yet species-dependent intracellular transformation patterns. In particular, tetravalent oxyanions (selenite and tellurite) underwent more rapid reduction and exhibited higher cellular accumulation than the corresponding hexavalent species in both algae, although the extent and kinetics of reduction differed markedly between species. Scanning and transmission electron microscopy demonstrated that selenium was immobilized as spherical elemental nanoparticles, whereas tellurium formed needle-like metallic nanorods within algal cells. Under hexavalent selenate exposure, higher-valent and organoselenium species remained detectable, and the formation of elemental selenium nanoparticles was limited. These results demonstrated speciation- and species-dependent intracellular transformation and accumulation of selenium and tellurium in unicellular algae, providing chemical speciation-based insights into algal chalcogen metabolism and detoxification processes.

## Graphical Abstract

**Graphical Abstract:**
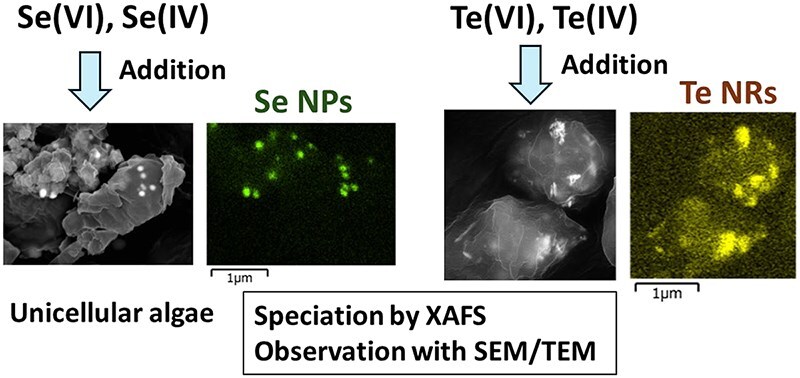

## Introduction

Selenium (Se) and tellurium (Te) are congeners belonging to group 16 of the periodic table and share similar electronic configurations and redox chemistry. Despite this chemical similarity, their biological roles differ markedly. Selenium is recognized as an essential trace element that is incorporated into selenoproteins and participates in redox regulation and antioxidant defence, whereas tellurium has no known biological function and is generally toxic to microorganisms, plants, and animals [1–3]. Understanding how these closely related chalcogen elements are taken up, transformed, and detoxified in living systems is therefore fundamental to elucidating the principles governing metal(loid) metabolism and speciation-dependent biological responses.

In aqueous and biological environments, selenium and tellurium predominantly occur as oxyanions in multiple oxidation states, including selenate [Se(VI)], selenite [Se(IV)], tellurate [Te(VI)], and tellurite [Te(IV)]. The oxidation state strongly influences transport pathways, reduction kinetics, and intracellular fate. Selenium, owing to its close chemical resemblance to sulphur, can be taken up *via* sulphate transporters and metabolized through sulphur-related pathways, leading to the formation of selenoamino acids, volatile organoselenium compounds, or elemental selenium [2]. In contrast, although the reduction of tellurite to elemental tellurium has been reported in bacteria and some eukaryotes [3], the detailed intracellular speciation changes and reduction pathways of tellurium remain considerably less understood. Although sulphur, selenium, and tellurium are congeners, they exhibit pronounced differences in essentiality, toxicity, and metabolic integration, making comparative studies of selenium and tellurium particularly valuable for understanding chalcogen biochemistry.

Beyond their biological significance, selenium and tellurium are technologically important elements. Selenium is widely used in photoconductive materials, glass processing, and catalytic systems [4–6], while tellurium is indispensable for solar cells, thermoelectric devices, and advanced electronic materials [7–9]. Both elements are classified as rare metals, and sustainable recovery and recycling strategies are increasingly required in light of resource limitations and environmental considerations. In this context, environmentally benign processes that exploit the physiological functions of microorganisms and plants for metal(loid) transformation have attracted growing attention [10, 11]. Numerous studies have demonstrated that algae, bacteria, fungi, and higher plants can reduce metal ions such as gold and silver to produce elemental nanoparticles and nanorods [12–16]. For selenium and tellurium, exposure to oxyanions has been shown to result in cellular accumulation followed by reduction to elemental nanoparticles or nanorods [17–21].

Among microalgae, the unicellular green alga *Chlamydomonas reinhardtii* has been reported to take up selenate and selenite and reduce them *via* pathways linked to sulphur metabolism, producing elemental selenium and volatile selenium species [22]. In contrast, exposure of *C. reinhardtii* to tellurite has been shown to induce the formation of intracellular tellurium nanorods [23]. We have previously reported the morphological and crystallographic characteristics of tellurium nanorods formed in *Pseudococcomyxa simplex* using transmission electron microscopy and electron diffraction [24]. These findings suggest that unicellular algae possess the ability to reduce chalcogen oxyanions and convert them into distinct elemental nanostructures.

However, several important issues remain unresolved. In particular, the intracellular chemical speciation of tellurium during uptake, reduction, and accumulation remains poorly understood. Moreover, systematic comparisons between selenium and tellurium under identical experimental conditions—especially with respect to oxyanion oxidation state and algal species—are scarce. Although elemental nanoparticle formation has been reported, direct spectroscopic evidence linking oxidation state-dependent reduction to specific intracellular chemical species and nanostructures remains limited. Speciation-resolved analyses are therefore critical for clarifying how closely related chalcogens diverge in their intracellular transformation and detoxification pathways. Chromatographic techniques such as high-performance liquid chromatography coupled with inductively coupled plasma mass spectrometry (ICP–MS) are widely used for speciation analysis; however, they generally require extraction and separation procedures, which may alter labile intracellular species. In contrast, X-ray absorption fine structure (XAFS) analysis enables nondestructive, *in situ* evaluation of chemical speciation and local coordination environments without chemical extraction, making it particularly suitable for investigating intracellular transformation processes. To elucidate such intracellular transformation mechanisms, experimental studies on microalgae often employ concentrations higher than environmental levels to detect changes in chemical form and reductions in metabolites.

In this study, we aimed to elucidate how selenium and tellurium oxyanions are transformed intracellularly in unicellular algae and how these transformations lead to the formation of elemental nanostructures. Two green microalgae, *C. reinhardtii* and *P. simplex*, were exposed to selenium and tellurium oxyanions in different oxidation states [Se(VI), Se(IV), Te(VI), and Te(IV)]. By combining quantitative analysis with XAFS to determine oxidation state and local coordination environment, and electron microscopy [scanning electron microscopy (SEM) and TEM] to directly visualize intracellular nanostructures and elemental distributions, we systematically examined the influence of oxidation state and algal species on accumulation, reduction behaviour, chemical speciation, and the formation of elemental nanostructures. Through this integrated speciation–structure approach, we sought to clarify both the common and distinct intracellular transformation pathways of these congeneric chalcogen elements in biological systems.

## Methods

### Cultivation of unicellular algae

The unicellular green algae *C. reinhardtii* (NIES-2235) and *P. simplex* (NIES-2713) were obtained from the NIES Collection (National Institute for Environmental Studies, Japan). Cells were cultivated in liquid suspension culture in 1000 mL marine flasks specifically designed for algal growth under ambient temperature conditions (22°C–26°C) with an illumination intensity of 3000–6000 lux, following previously reported procedures [24, 25]. Subculturing was performed every two weeks to maintain stable growth conditions.

### Preparation of selenium and tellurium solutions

Sodium selenate (Na_2_SeO_4_, KANTO CHEMICAL, 37266-32), potassium selenite (K_2_SeO_3_, KANTO CHEMICAL, 32393-32), telluric acid (H_6_TeO_6_, KANTO CHEMICAL, 40008-30), and potassium tellurite (K_2_TeO_3_, KANTO CHEMICAL, 32410-30) were dissolved in ultrapure water to prepare aqueous exposure solutions corresponding to 100 mg L^−1^ as Se or 100 mg L^−1^ as Te, respectively. Concentrations were calculated on an elemental basis rather than as compound mass. The pH of each solution was adjusted to 6–7 using hydrochloric acid.

### Exposure experiments and sample preparation

Algal cultures (240 mL) were centrifuged to harvest cells, and the biomass was resuspended in 30 mL of selenium or tellurium solution at 100 mg L⁻¹ as Se or 100 mg L⁻¹ as Te. This concentration was selected to allow reliable evaluation of intracellular chemical speciation and is consistent with concentration ranges commonly used in microalgal studies investigating chalcogen accumulation and transformation [17]. Incubation was carried out under continuous horizontal shaking (TAITEC NR-3) in an incubator (TOMY CLE-303) at 23.0°C, relative humidity 70%–80%, and a 16 h light (12 000 lx)/8 h dark (400 lx) cycle. Exposure experiments were conducted over incubation periods ranging from 6 to 168 h, depending on the experimental series.

After exposure, algal cells were collected by centrifugation, washed three times with ultrapure water to remove externally adsorbed species, and freeze-dried for 24 h. Portions of the dried samples were used for electron microscopy. The remaining samples were homogenized in an agate mortar, and ~10 mg of each sample was pressed into 5 mm diameter pellets for XAFS analysis.

### Determination of selenium and tellurium concentrations in algal cells by X-ray fluorescence analysis

Elemental concentrations of Se and Te in algal biomass were determined using an energy-dispersive X-ray fluorescence spectrometer equipped with a polarization optics system (Epsilon 5, Malvern Panalytical). A gadolinium X-ray tube and a germanium semiconductor detector were employed in a Cartesian optical configuration. Zirconium and cerium oxide (CeO_2_) secondary targets were used to efficiently excite the Se and Te Kα lines. Measurement conditions were 80 kV and 6 mA for Se, and 100 kV and 6 mA for Te, with an acquisition time of 300 s.

Calibration curves were constructed to minimize matrix effects by preparing standards composed of cellulose powder (Wako, 036-22225) mixed with sodium selenate (for Se) or metallic tellurium powder (ALDRICH, 266418-25 G) (for Te). Net X-ray fluorescence intensities were normalized to the Compton scattering intensity of the secondary target prior to calibration.

### Determination of residual selenium and tellurium concentrations in solutions by ICP–OES

Following centrifugation, the supernatant solution was collected without acid digestion. The sample was diluted as required and analysed directly by inductively coupled plasma optical emission spectrometry (ICP–OES; Shimadzu ICPE-9820).

Operating conditions were: RF power 1.2 kW, plasma gas flow 10.0 L min⁻¹, auxiliary gas flow 0.60 L min⁻¹, and carrier gas flow 0.70 L min^−1^. Emission lines at 196.090 nm (Se) and 238.578 nm (Te) were selected to minimize spectral interference. Yttrium was added and used as an internal standard.

To evaluate potential elemental loss during incubation, mass balance analysis was performed by summing the amount remaining in solution (residual concentration × solution volume) and the amount accumulated in biomass (cellular concentration × dry mass), and comparing the total with the initial amount added.

### XAFS analysis

Se K-edge XAFS measurements were performed at beamlines BL-9A and BL-12C of the Photon Factory (PF), High Energy Accelerator Research Organization (KEK), Japan. The incident X-ray beam was monochromatised using a Si(111) double-crystal monochromator. Transmission measurements were conducted with ionization chambers filled with N₂ (85%)–Ar (15%) and Ar (100%) for incident (*I*_0_) and transmitted (*I*) intensities, respectively. Fluorescence measurements were carried out using a seven-element silicon drift detector equipped with a Soller slit and an arsenic filter to suppress scattered X-rays and improve the signal-to-background ratio. Energy calibration was carried out using sodium selenate, with the peak position set to 8.9886 deg (12.655 keV).

Reference compounds (sodium selenate, potassium selenite, metallic selenium powder, and methylselenocysteine) were diluted with boron nitride (BN) and measured under identical conditions.

Te K-edge XAFS measurements were conducted at beamline NW-10A of the PF–Advanced Ring. The incident X-ray beam was monochromatised using a Si(311) double-crystal monochromator. Transmission measurements employed ionization chambers filled with Ar (100%) and Kr (100%), while fluorescence measurements were performed using a 19-element germanium semiconductor detector equipped with a Soller slit and a tin filter. Energy calibration was performed using telluric acid by aligning the peak position in the first derivative of the XAFS spectrum to 6.8324 deg (31.812 keV). Telluric acid, potassium tellurite, and metallic tellurium powder diluted with BN were used as reference compounds.

XAFS data were analysed using REX2000 software (Ver. 2.5.7, Rigaku, Japan). XANES analysis was performed by linear combination fitting (LCF) over the energy ranges 12 630–12 730 eV for Se [26] and 31 790–31 840 eV for Te [27] to estimate the relative fractions of chemical species with different oxidation states. The goodness of fit was evaluated using the *R*-factor defined as:


\begin{eqnarray*}
R = \frac{{\sum {{\left\{ {{\mu _{obs}}\left( E \right) - {\mu _{cal}}\left( E \right)} \right\}}^2}}}{{\sum {{\left\{ {{\mu _{obs}}\left( E \right)} \right\}}^2}}}
\end{eqnarray*}

EXAFS analysis was performed using *k*³-weighted χ(*k*) functions. The χ(*k*) data were Fourier transformed using appropriate window functions to obtain radial structure functions. Theoretical amplitudes and phase shift were calculated based on McKale’s theoretical tables. The agreement between experimental and calculated EXAFS oscillations was evaluated using the *R-*factor defined as:


\begin{eqnarray*}
R = \frac{{\sum {{\left\{ {{k^3}{\chi _{obs}}\left( k \right) - {k^3}{\chi _{\textit{calc}}}\left( k \right)} \right\}}^2}}}{{\sum {{\left\{ {{k^3}{\chi _{obs}}\left( k \right)} \right\}}^2}}}
\end{eqnarray*}

### Electron microscopy: scanning electron microscopy (SEM) and transmission electron microscopy (TEM)

Freeze-dried samples were mounted on conductive tape, coated with osmium, and then subjected to electron microscopy. Morphological features and elemental distributions were investigated by scanning and transmission electron microscopy. SEM observations were conducted using a field-emission scanning electron microscope (JSM-7100 TTL, JEOL, Tokyo, Japan) at 15 kV in backscattered electron mode. Electron mapping was performed using an Energy-dispersive X-ray spectroscopy (EDS) detector (X-Max 80, Oxford Instruments, Abingdon, UK).

TEM and scanning transmission electron microscopy (STEM) observations were performed using a JEOL JEM- F200 operated at 200 kV and an FEI Titan Cubed G2 60-300 operated at 300 kV. Freeze-dried samples were dispersed directly onto polymer-support copper grids and examined without chemical fixation, embedding, or ultrathin sectioning to minimize artifacts and preserve intracellular nanostructures.

## Results

### Accumulation behaviour and chemical speciation of selenium in algal cells

The concentrations of selenium accumulated in algal cells are summarized in Table 1. In both algal species, cellular selenium concentrations increased with increasing exposure time. However, the accumulation was strongly dependent on the oxidation state of the added selenium species.

**Table 1.** Selenium concentrations accumulated in *C. reinhardtii* and *P. simplex* after exposure to selenium oxyanions

Exposure time/h	Se concentration in algae (mg/kg DW)				
	Species	*C. reinhardtii*		*P. simplex*	
	Added	Se(VI)	Se(IV)	Se(VI)	Se(IV)
6		248	7200	15.2	129
24		650	25 800	64.3	811
96		877	37 800	237	5470
168		1080	42 600	295	13 200

In *P. simplex*, selenium concentrations reached 295 ppm after 168 h of selenate exposure, whereas selenite exposure resulted in a markedly higher accumulation (13,200 ppm). A similar but more pronounced trend was observed in *C. reinhardtii*, where selenite exposure led to substantially higher accumulation than selenate exposure. After 168 h, selenium concentrations reached 1080 ppm under selenate treatment and 42 600 ppm under selenite treatment.

**Figure 1:**
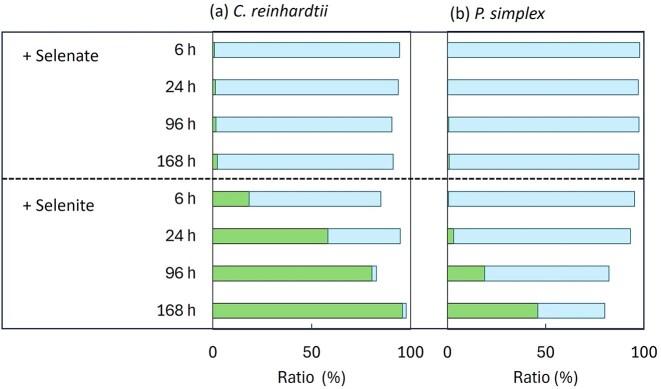
Distribution of selenium between algal cells and the residual solution after exposure to selenium oxyanions. (a) *Chlamydomonas reinhardtii* and (b) *P. simplex* were exposed to selenate [Se(VI)] or selenite [Se(IV)] for 6, 24, 96, and 168 h. The proportions of selenium accumulated in algal cells (filled bar) and remaining in the solution (unfilled bar) were calculated based on the total selenium mass.

The distribution of selenium between algal cells and residual solution is shown in Fig. 1. Under selenate treatment, most of the added selenium remained in solution throughout the exposure period. In contrast, selenite treatment led to a progressive transfer of selenium from the solution to algal cells, particularly after prolonged exposure (168 h). This behaviour was consistent in both species.

Although both algal species showed higher accumulation for selenite, pronounced interspecies differences were evident from the early stages of exposure. After 6 h of selenite exposure, selenium concentrations were 129 ppm in *P. simplex* and 7200 ppm in *C. reinhardtii*, corresponding to ~55-fold higher accumulation in *C. reinhardtii*. After 168 h of exposure, 96% of the added selenium was recovered in *C. reinhardtii* biomass (Fig. 1).

**Figure 2:**
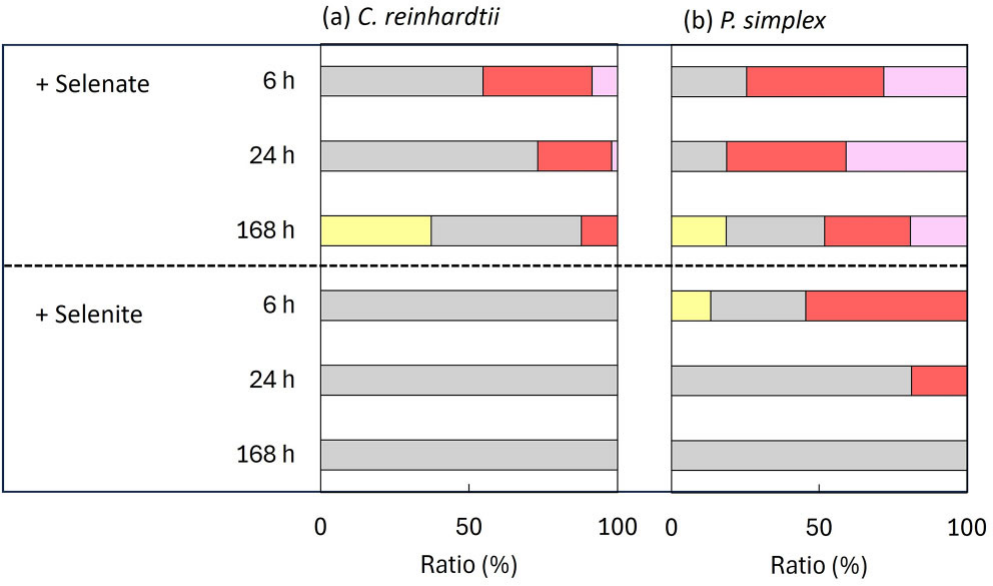
Relative proportions of selenium species with different oxidation states in algal cells, determined by LCF of Se K-edge XANES spectra. Oxidation states are colour-coded as follows: Se(−II) (unfilled), Se(0) (grey-filled), Se(IV) (striped), and Se(VI) (solid-filled). Reference compounds used for LCF were elemental selenium powder (R1), potassium selenite (R2), sodium selenate (R3), and methylselenocysteine (R4).

The oxidation state distribution of intracellular selenium was evaluated by Se K-edge XANES analysis (Fig. S1), and LCF results are summarized in Fig. 2. Selenium species corresponding to Se(-II), Se(0), Se(IV), and Se(VI) were quantified using reference compounds.

For *C. reinhardtii* exposed to selenate [Se(VI)], ~37% of accumulated selenium was present as Se(IV) and ∼55% as Se(0) after 6 h. After 168 h, the selenium speciation further shifted to reduced forms, with ∼37% Se(-II), ∼51% Se(0), and ∼12% Se(IV). In contrast, under selenite [Se(IV)] exposure, selenium accumulated in *C. reinhardtii* was almost entirely present as Se(0) within 6 h and remained predominantly Se(0) up to 168 h (Fig. 2a).

In *P. simplex*, selenium was also reduced after uptake, but the proportion of Se(0) was consistently lower than in *C. reinhardtii* (Fig. 2b). For all samples, the *R*-factors (%) for LCF analysis ranged from 0.028 to 0.095, indicating satisfactory agreement between experimental and fitted data.

**Figure 3:**
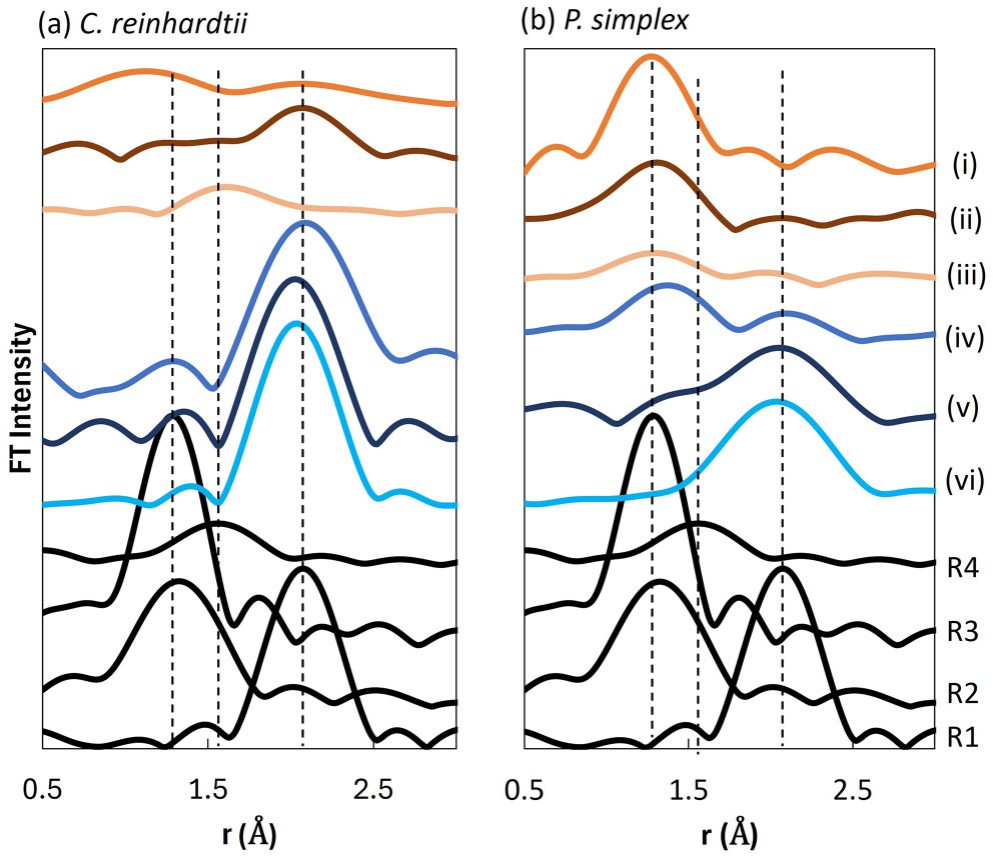
Fourier-transformed Se K-edge EXAFS data (not phase-corrected) of (a) *C. reinhardtii* and (b) *P. simplex* after exposure to selenate and selenite, together with reference compounds. EXAFS data (i), (ii), and (iii) correspond to samples exposed to selenate [Se(VI)] for 6, 24, and 168 h, respectively, whereas (iv), (v), and (vi) correspond to samples exposed to selenite [Se(IV)] for the same incubation times. Reference data are shown for comparison: elemental selenium powder (R1), potassium selenite (R2), sodium selenate (R3), and methylselenocysteine (R4). Dashed vertical lines indicate the approximate positions of characteristic Se–Se, Se–O, and Se–C scattering contributions.

The Se K-edge EXAFS-derived radial structure functions are shown in Fig. 3 together with reference compounds of metallic selenium powder (R1), potassium selenite (R2), sodium selenate (R3), and methylselenocysteine (R4). Distinct contributions assignable to Se–Se, Se–O, and Se–C interactions were identified (fitting results are summarized in Table S1).

In selenate-treated *C. reinhardtii* [Fig. 3a(i–iii)], the Se–O contributions were already weak after 6 h. A broad Se–Se feature appeared after 24 h, and after 168 h, an additional contribution attributable to Se–C coordination became evident. In contrast, selenite-treated *C. reinhardtii* [Fig. 3a(iv–vi)] showed clear Se–Se interactions from 6 h onward, which sharpened with time, approaching the local structure of metallic selenium.

In *P. simplex*, selenate exposure initially produced a prominent Se–O contribution (6 h), which progressively decreased and became minimal after 168 h [Fig. 3b(i–iii)]. Under selenite exposure [Fig. 3b(iv–vi)], Se–O contributions were initially present but diminished over time, while Se–Se interactions became dominant.

### Accumulation behaviour and chemical speciation of tellurium in algal cells

Tellurium concentrations in algal cells are summarized in Table 2. In both algal species, cellular tellurium concentrations increased with exposure time and were consistently higher under tellurite [Te(IV)] exposure than under tellurate [Te(VI)] exposure.

**Table 2.** Tellurium concentrations accumulated in *C. reinhardtii* and *P. simplex* after exposure to tellurium oxyanions

Exposure time/h	Te concentration in algae (mg/kg DW)				
	Species	*C. reinhardtii*		*P. simplex*	
	Added	Te(VI)	Te(IV)	Te(VI)	Te(IV)
6		116	1830	640	1300
24		142	7540	1270	2960
96		613	6120	3780	5790
168		1040	48 200	5320	9400

**Figure 4:**
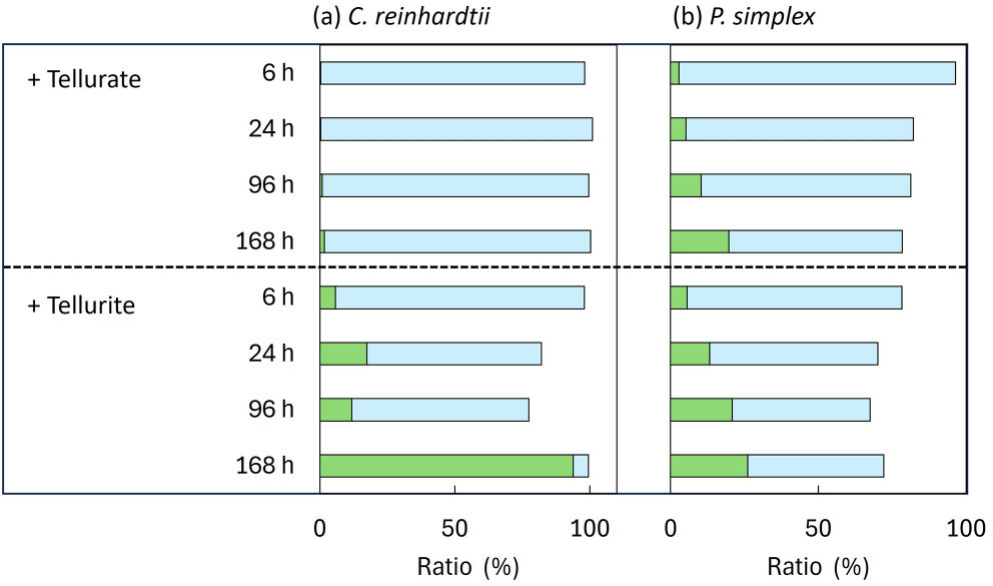
Distribution of tellurium between algal cells and the residual solution after exposure to tellurium oxyanions. (a) *Chlamydomonas reinhardtii* and (b) *P. simplex* were exposed to tellurate [Te(VI)] or tellurite [Te(IV)] for 6, 24, 96, and 168 h. The proportions of tellurium accumulated in algal cells (filled) and remaining in the solution (unfilled) were calculated based on the total tellurium mass.

The distribution of tellurium between algal cells and solution is shown in Fig. 4. In *C. reinhardtii*, most tellurate remained in solution, whereas tellurite exposure resulted in substantial accumulation in algal biomass after 168 h. Similar behaviour was observed in *P. simplex*.

**Figure 5:**
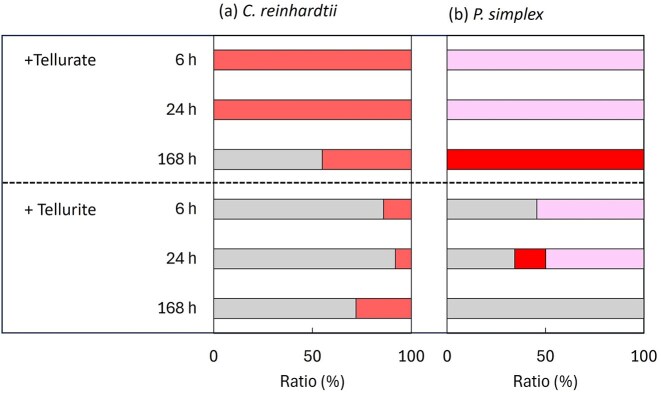
Relative proportions of tellurium species with different oxidation states in algal cells, determined by LCF of Te K-edge XANES spectra. Oxidation states are coded as follows: Te(0) (grey-filled), Te(IV) (unfilled), and Te(VI) (solid-filled). Reference compounds used for LCF were metallic tellurium powder (R1), potassium tellurite (R2), and telluric acid (R3).

The oxidation state distribution of tellurium in algal cells was evaluated by Te K-edge XANES analysis (Fig. S2), and the LCF results are summarized in Fig. 5. In *C. reinhardtii*, tellurate was rapidly reduced to Te(IV) within 6 h, and after 168 h, ~55% of accumulated tellurium was present as Te(0) (Fig. 5a). Tellurite exposure resulted in even faster reduction, with ∼86% of Te(0) already present after 6 h.

In *P. simplex* (Fig. 5b), tellurate remained predominantly Te(VI) during the initial 6–24 h, with partial reduction to Te(IV) observed only after 168 h. Under tellurite exposure, ∼46% Te(0) was present after 6 h, increasing to nearly complete reduction after 168 h. *R*-factors (%) for the LCF analysis ranged from 0.019 to 0.069.

**Figure 6:**
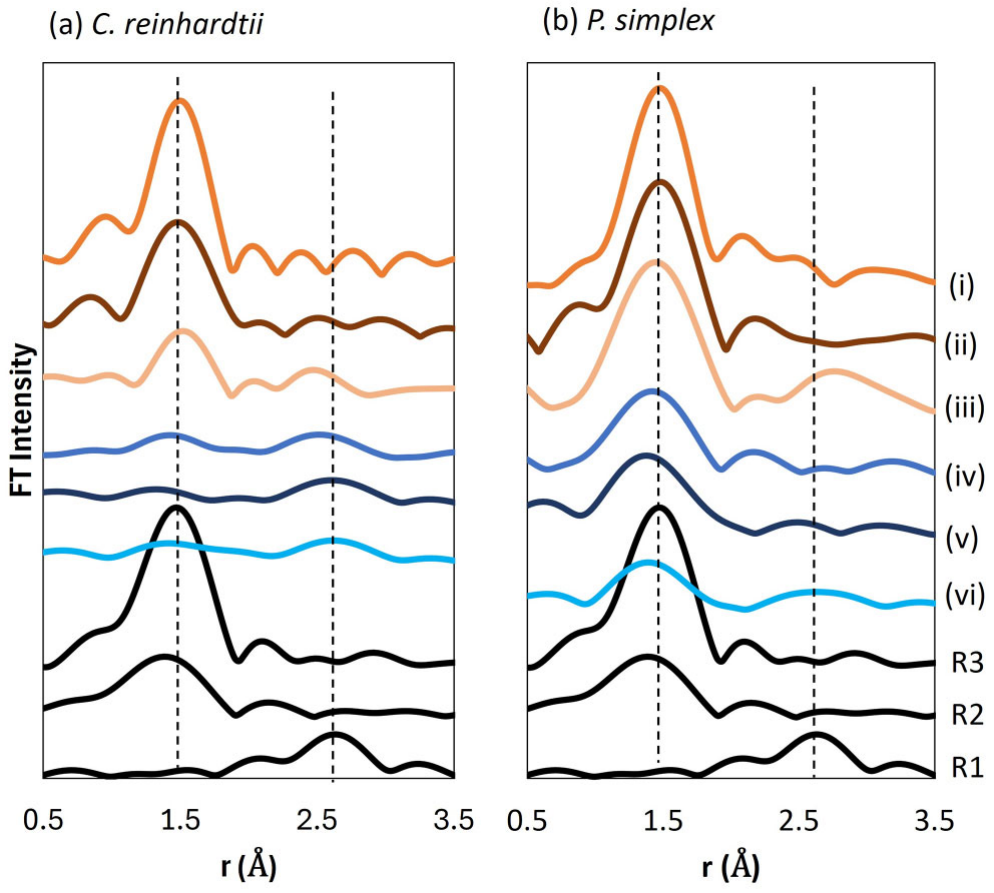
Fourier-transformed Te K-edge EXAFS data (not phase-corrected) of (a) *C. reinhardtii* and (b) *P. simplex* after exposure to tellurate and tellurite, together with reference compounds. EXAFS data (i), (ii), and (iii) correspond to samples exposed to tellurate [Te(VI)] for 6, 24, and 168 h, respectively, whereas (iv), (v), and (vi) correspond to samples exposed to tellurite [Te(IV)] for the same incubation times. Reference data are shown for comparison: metallic tellurium powder (R1), potassium tellurite (R2), and telluric acid (R3). Dashed vertical lines indicate the approximate positions of characteristic Te–Te and Te–O scattering contributions.

The Te K-edge EXAFS radial structure functions are shown in Fig. 6, together with reference compounds of metallic tellurium powder (R1), potassium tellurite (R2), and telluric acid (R3). Distinct contributions assignable to Te–Te and Te–O interactions were identified (fitting results are summarized in Table S2).

Tellurate-treated samples [Fig. 6a(i–iii)] exhibited clear Te–O contributions at early time points, which decreased with increasing exposure time. Tellurite-treated *C. reinhardtii* [Fig. 6a(iv–vi)] showed weak Te–O contributions and distinct Te–Te interactions even at 6 h. In *P. simplex* (Fig. 6b), Te–O features were more persistent but gradually gave way to Te–Te interactions.

### SEM and TEM observations of selenium- and tellurium-treated algal cells

**Figure 7:**
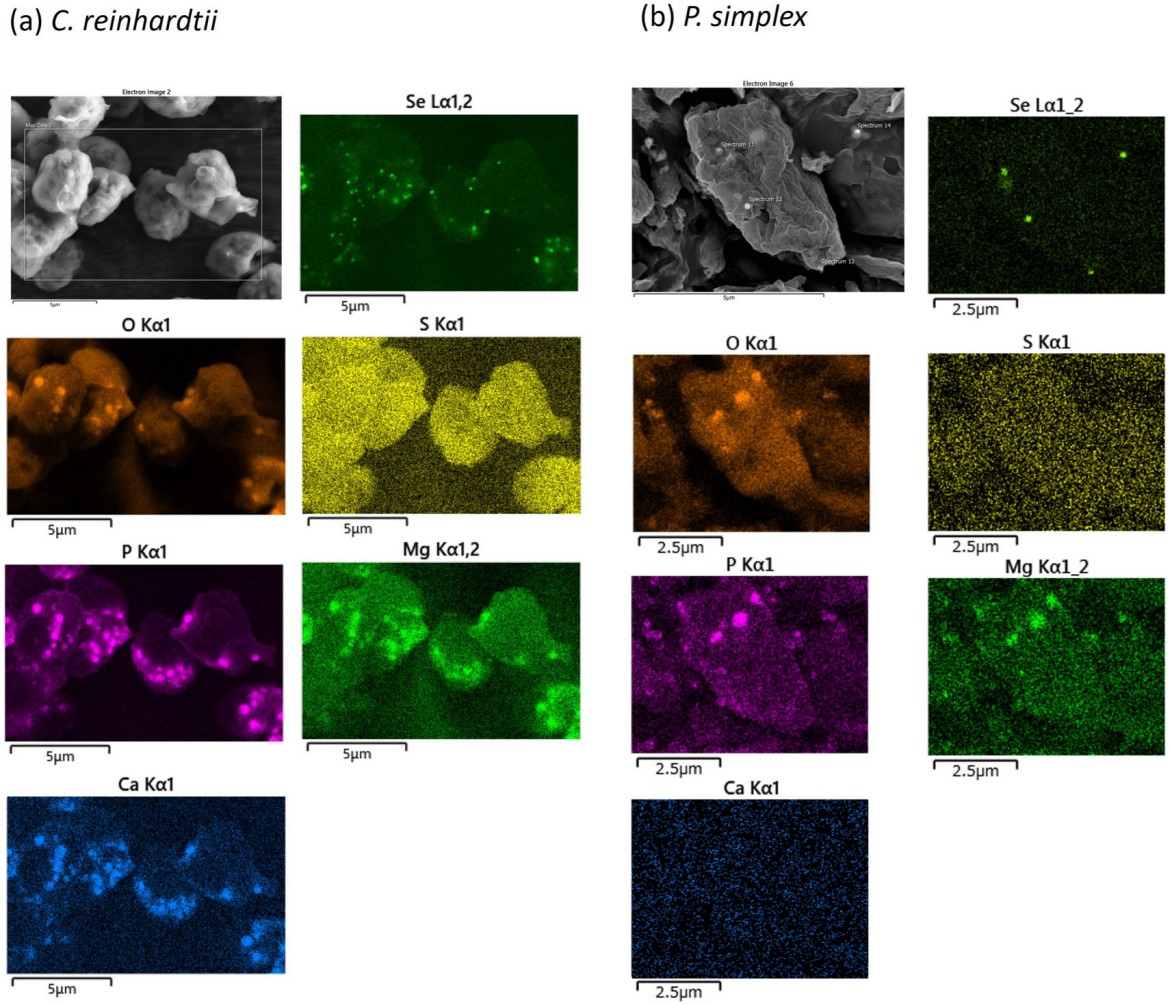
SEM images of unicellular algae after exposure to selenite. (a) *Chlamydomonas reinhardtii* and (b) *P. simplex* after 24 h exposure to selenite. Spherical nanoparticles with diameters of ~200–300 nm were observed in both algal species. EDS mapping revealed a strong spatial correlation between the nanoparticles and selenium signals.

SEM images of algal cells exposed to selenite for 24 h are shown in Fig. 7. Spherical nanoparticles with diameters of ~200–300 nm were observed in both algal species. EDS confirmed that these particles consisted predominantly of selenium. No such nanoparticles were observed under selenate exposure.

**Figure 8:**
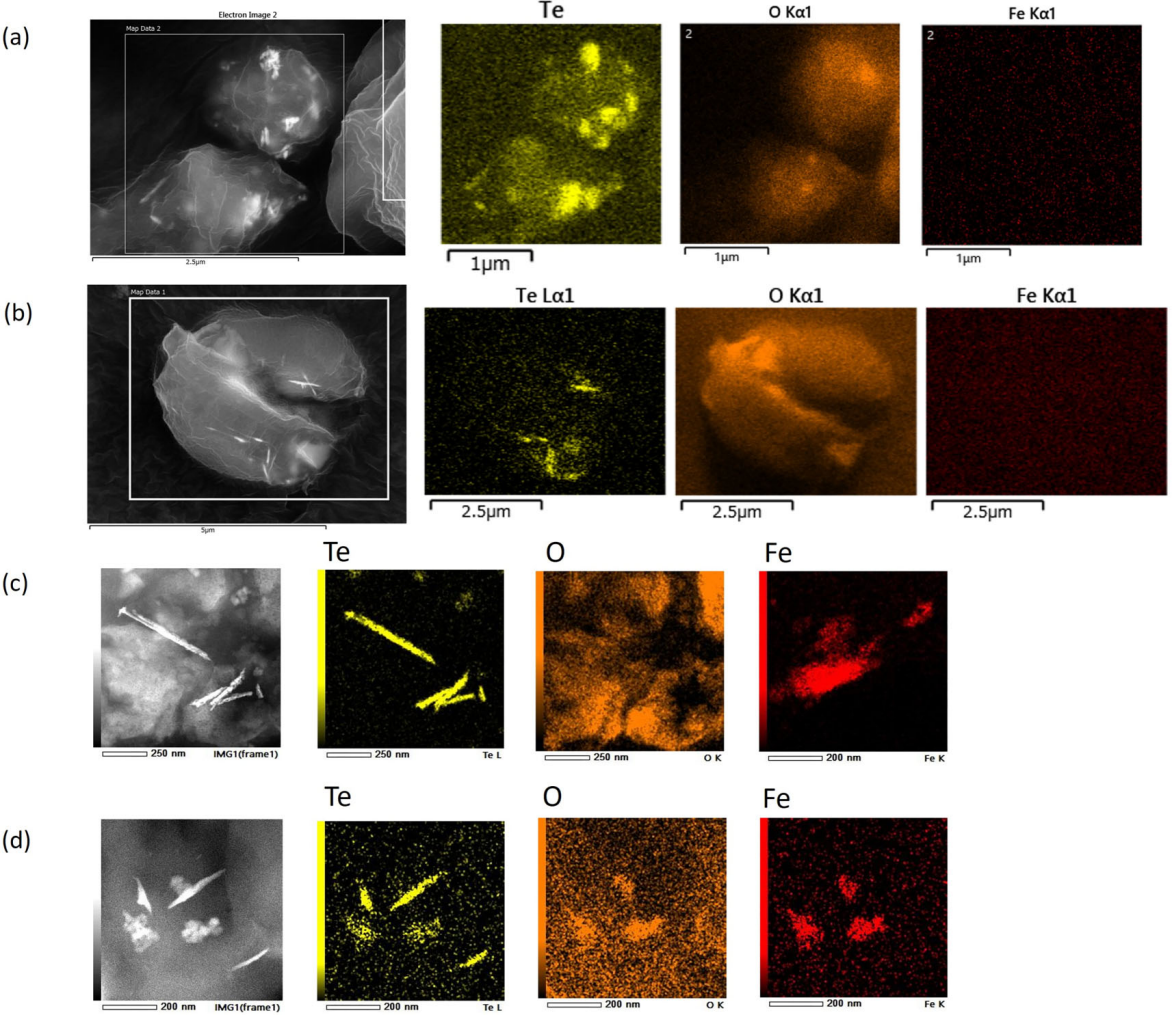
SEM and STEM images with elemental mapping of unicellular algal cells after exposure to tellurium oxyanions. (a, b) SEM images acquired at an accelerating voltage of 15 kV. (c, d) STEM images and corresponding STEM–EDS elemental maps acquired at 200 kV using a JEOL JEM-F200. (a) *Chlamydomonas reinhardtii*and (b) *P. simplex* exposed to tellurite for 24 h. (c) *Pseudococcomyxa simplex* exposed to tellurite for 168 h, and (d) *P. simplex* exposed to tellurate for 168 h. Needle-like tellurium nanorods were observed inside the algal cells under both tellurite and tellurate exposure conditions. Elemental mapping demonstrates that tellurium is localized within the cells. Panels (b) and part of the STEM/EDS datasets have been reported previously [24].

SEM and STEM images following tellurium exposure are shown in Fig. 8. After 24 h of tellurite exposure, needle-like nanostructures were observed in both *C. reinhardtii* (Fig. 8a) and *P. simplex* (Fig. 8b). After 168 h, elongated nanorods were clearly observed in *P. simplex* under both tellurite (Fig. 8c) and tellurate (Fig. 8d) exposure.

The algal cells had an approximate thickness of 3 μm, and STEM observations were performed at the cell periphery. This configuration enabled direct transmission imaging of intracellular structure without chemical pretreatment, sectioning, or extraction procedures. Under these conditions, needle-like nanostructures measuring ~200–600 nm in length and ∼30 nm in width were observed within the cell interior.

STEM-EDS elemental mapping demonstrated strong tellurium enrichment and minimal oxygen contribution, consistent with metallic tellurium. Additional TEM and STEM-EDS data for 7-day tellurite exposure are provided in Fig. S3 and further confirm intracellular localisation.

Selected-area electron diffraction patterns in intracellular nanorods are shown in Fig. S4. They indicate the crystalline nature of the intracellular nanorods. The data obtained from the intracellular nanorods were attributed to diffraction spots corresponding to the (111) and (003) planes of trigonal metallic tellurium, confirming the crystalline nature of the nanorods.

## Discussion

XAFS analyses together with electron microscopy revealed that selenium and tellurium oxyanions taken up by *C. reinhardtii* and *P. simplex* underwent intracellular reduction irrespective of the oxidation state of the oxyanions supplied (VI or IV). Nevertheless, both the extent and kinetics of reduction, as well as accumulation behaviour, differed markedly depending on the chemical species added and on algal species, suggesting that speciation-dependent uptake and transformation pathways operate in these unicellular algae.

### Effect of selenium oxidation state on intracellular transformation and nanoparticle formation

For selenium, selenite [Se(IV)] resulted in substantially higher cellular accumulation than selenate [Se(VI)] in both algae. This difference was consistently reflected across mass balance results, XANES LCF analysis, EXAFS data, and microscopy observations. Under selenite exposure, Se–Se interactions were already evident at an early stage in *C. reinhardtii* and became progressively more pronounced with time, consistent with rapid conversion to elemental selenium. In contrast, under selenate exposure, the Se–Se feature remained weak and broad in both algae, indicating that although Se(0) was formed, it did not develop into a well-defined and locally ordered elemental phase.

XANES LCF analysis provided quantitative support for these trends. Selenite-treated cells were dominated by Se(0) from the early phase, whereas selenate-treated cells retained contributions from Se(VI) together with organic selenium-related components, while also containing appreciable Se(0). However, this Se(0) did not accumulate as a sufficiently concentrated and structurally ordered phase to enable detectable nanoparticle formation under the present conditions. These observations indicate that reduction of selenate occurs but does not necessarily lead to extensive elemental selenium aggregation.

SEM observations were fully consistent with the speciation results: spherical nanoparticles (200–300 nm) were observed after 24 h in selenite-treated samples, whereas no distinct nanoparticles were detected in selenate-treated samples even after prolonged exposure. Taken together, these findings demonstrate that selenite is rapidly reduced and immobilized as elemental selenium in a form that readily nucleates and grows into microscopically observable nanoparticles, whereas selenate-derived Se(0) remains more dispersed and/or metabolically partitioned.

This speciation-dependent behaviour aligns with established models of selenium metabolism in plants and algae, in which selenate is taken up *via* sulphate transporters and reduced through ATP sulphurylase-dependent steps, leading to incorporation into organic selenium species (e.g. selenomethionine) and/or conversion to Se(0) [28, 29]. The observation of Se–C contributions in EXAFS and organic selenium components in XANES LCF for selenate-treated samples is consistent with the involvement of such metabolic routes. In contrast, selenite requires fewer reductive steps and is known to be more readily converted to Se(0) [30], providing a plausible basis for the rapid reduction and high accumulation observed here.

Notably, selenium nanoparticles were detected within 24 h under selenite exposure. Compared with the previous report in which selenium nanoparticle biosynthesis in *C. reinhardtii* required 6 days of exposure to sodium selenite in an ApScl-overexpressing strain [31], the present results demonstrate that nanoparticle formation can proceed on a shorter timescale under nongenetically modified conditions. In addition, biosynthesis of selenium nanoparticles has been reported using extracts of *Spirulina platensis* [32] and *Nannochloropsis oceanica* [33], whereas the present study demonstrates that intact algal cells can drive both reduction and intracellular immobilization without the addition of external reductants or extracts.

Reduction and immobilization of selenium as insoluble Se(0) has been discussed as a detoxification-related outcome in other biological systems. For example, insoluble selenium nanoparticles formed upon selenate exposure in *Aspergillus niger* have been regarded as inert and less toxic forms of selenium [34, 35]. The present findings suggest that in unicellular algae, reduction and nanoparticle formation may similarly contribute to effective intracellular retention and immobilization, thereby supporting high accumulation under conditions favouring elemental selenium formation.

### Tellurium reduction and intracellular nanorod formation

For tellurium, both tellurate [Te(VI)] and tellurite [Te(IV)] underwent progressive reduction in algal cells, as evidenced by decreasing Te–O contributions and emerging Te–Te interactions in EXAFS over time. XANES LCF analysis likewise indicated that oxyanion-derived components dominated at early time points, whereas the Te(0) contribution increased substantially by 168 h. These observations demonstrate that tellurium is reduced intracellularly in a stepwise manner, ultimately yielding elemental tellurium.

Electron microscopy provided direct evidence that this chemical transformation is accompanied by intracellular formation of needle-like tellurium nanorods, and electron diffraction confirmed a trigonal metallic tellurium phase rather than tellurium oxides. The ability to identify intracellular metallic nanorods without chemical pretreatment is particularly valuable for discussing biological immobilization processes, because conventional extraction-based approaches may alter labile intermediates or disrupt spatial relationships between nanostructures and cellular components.

An important feature emerging from the combined dataset is that reduction and nanorod formation proceed on different timescales. Although Te–Te interactions indicative of reduced tellurium were detected by EXAFS at earlier stages, rod-like crystals were not observed in short-term TEM observations. This implies that tellurium may first be reduced to Te(0)-containing species, while subsequent crystallization and anisotropic growth into nanorods requires additional time (days). Thus, chemical reduction (speciation change) and nanorod formation (crystal growth) should be considered as separable processes.

The dimensions of tellurium nanorods produced by physical and chemical syntheses span from hundreds of nanometres to several micrometres [36, 37], whereas extracellular bacterial biosynthesis by *Stenotrophomonas maltophilia* and *Ochrobactrum anthropi* has been reported to yield nanorods of ∼100–300 nm [38, 39]. The intracellular nanorods observed here (typically 200–600 nm in length and ∼30 nm in width) are comparable in size to those produced by bacteria and are generally shorter than those obtained by many chemical syntheses, suggesting that biological constraints within cells may limit rod growth.

In bacterial systems, the formation of tellurium nanomaterials often requires anaerobic conditions and/or the addition of external reductants [40–42]. For example, in *Escherichia coli*, reduction of tellurite and extracellular nanorod formation were enhanced under anaerobic conditions in the presence of quinone redox mediators such as lawsone and menadione [43]. In contrast, the present study demonstrates that algal cells can generate tellurium nanorods under aerobic conditions without exogenous reductants, consistent with the notion that algal intracellular reductants and/or enzymatic systems contribute to the reduction of tellurate and tellurite [23].

Tellurium is highly toxic to many organisms [3], and previous studies addressing detoxification mechanisms have been dominated by bacterial research [44]. The observed conversion of tellurium oxyanions to insoluble Te(0) nanorods supports the possibility that unicellular algae also mitigate tellurium toxicity through reduction and intracellular immobilization. Reports of biogenic tellurium nanorods in garlic [45] and in *C. reinhardtii* upon tellurate exposure [23] further support a detoxification-related interpretation, where higher tellurate concentrations increased the proportion of insoluble Te(0). The present results are consistent with these observations and extend them by providing direct evidence of speciation during intracellular transformation.

### Species-dependent differences in reduction and accumulation

Comparative analysis of the two algal species revealed clear differences in accumulation and reduction behaviour for both selenium and tellurium. For selenium, particularly under selenite exposure, *C. reinhardtii* exhibited much higher accumulation and earlier emergence of Se–Se interactions than *P. simplex*. XANES LCF likewise indicated higher early contributions of Se(0) in *C. reinhardtii*, while *P. simplex* tended to retain Se–O and/or organic selenium-related contributions for longer periods. These results suggest that *C. reinhardtii* possesses a greater capacity for selenium reduction and intracellular immobilization under the present conditions.

*Chlamydomonas reinhardtii* is a well-established model alga for which sulphur-related transport and reductive pathways have been intensively studied, and it may therefore take up selenium efficiently as a sulphur analogue. By contrast, fewer mechanistic studies are available for *P. simplex*, and differences in transport and/or intracellular reductive capacity may underlie the observed kinetic differences. Consistent with this, *P. simplex* retained Se–O features for longer under selenate exposure, suggesting limited conversion to elemental selenium compared with *C. reinhardtii*.

For tellurium, Te K-edge XAFS and XANES LCF indicated that *C. reinhardtii* exhibited earlier formation of Te(0)-related components, whereas *P. simplex* retained Te–O coordination for longer. Nonetheless, with increasing exposure time both algae converged towards a Te–Te-dominated local structure, and metallic tellurium nanorods were confirmed by electron microscopy. Thus, species-dependent differences appear to be reflected more strongly in the kinetics and intermediates of reduction and immobilization than in the final elemental product.

The observed species-dependent differences in accumulation efficiency, reduction kinetics, and chemical speciation are unlikely to arise from entirely distinct uptake mechanisms, but rather from variations in the efficiency and regulation of fundamentally shared pathways. Selenium uptake in algae is generally mediated by transport systems associated with sulphur metabolism, and such systems are expected to be conserved across different algal species [28–30].

Therefore, the differences observed between *C. reinhardtii* and *P. simplex* are more plausibly attributed to differences in uptake rates, transporter activity, intracellular redox capacity, and subsequent metabolic processing, rather than to the presence of fundamentally different mechanisms. These differences may influence the balance between reduction, metabolic incorporation, and immobilization, leading to the observed variations in accumulation efficiency and chemical speciation.

Importantly, the systematic comparison of two algal species under identical experimental conditions in this study enabled us to distinguish shared transformation behaviour from species-dependent variations. However, the present study does not directly identify the molecular mechanisms responsible for these interspecies differences. Further investigations focusing on transporter activity, enzymatic reduction pathways, and intracellular redox processes will be required to elucidate the underlying mechanisms in detail.

### Similarities and contrasts between selenium and tellurium intracellular fates

A key outcome of this study is that both selenium and tellurium are taken up as oxyanions and undergo intracellular reduction, yet their ultimate fates differ substantially. For selenium, selenite exposure promoted rapid formation of elemental selenium nanoparticles, whereas under selenate exposure, elemental nanoparticle formation was not apparent and XANES LCF indicated persistence of Se(VI) and organic selenium components. This suggests that selenate is preferentially partitioned into metabolic pathways that form organic selenium rather than being fully immobilized as elemental selenium.

In contrast, tellurium was ultimately reduced to Te(0) and immobilized as insoluble nanorods irrespective of the initial oxidation state supplied. This contrast likely reflects the strong chemical and biochemical similarity of selenium to sulphur, allowing selenium to be incorporated into established sulphur-related metabolic networks, whereas tellurium is less readily incorporated into such metabolism and is instead processed predominantly via reduction and immobilization as a detoxification strategy. Together, the combined speciation and imaging data support the view that selenium behaves largely as an element subject to metabolic processing, whereas tellurium is treated primarily as a toxicant that is detoxified and immobilized intracellularly.

## Conclusion

In this study, the accumulation behaviour and intracellular chemical transformations of selenium and tellurium oxyanions were systematically investigated in two unicellular algae, *C. reinhardtii* and *P. simplex*, using XAFS analysis combined with electron microscopy. Selenium and tellurium were reduced intracellularly irrespective of their initial oxidation states [Se(VI)/Se(IV) and Te(VI)/Te(IV)]; however, the extent of reduction, accumulation efficiency, and final chemical speciation strongly depended on the chemical speciation of the oxyanions and the algal species.

Selenite [Se(IV)] was rapidly reduced to elemental selenium [Se(0)], which accumulated as spherical nanoparticles, whereas selenate [Se(VI)] was partly retained as higher-valent and/or organoselenium species, resulting in limited formation of Se(0) nanoparticles. In contrast, both tellurate [Te(VI)] and tellurite [Te(IV)] were ultimately reduced to elemental tellurium [Te(0)] and immobilized as intracellular tellurium nanorods. Notably, these nanostructures were formed under aerobic conditions without the addition of external reducing agents, indicating that endogenous algal components directly mediate chalcogen reduction and immobilization.

These results demonstrate that unicellular algae exhibit differential intracellular transformation of chalcogen oxyanions depending on their chemical speciation, leading to distinct metabolic or detoxification outcomes. The combined speciation analysis and morphological observations provide new insights into the contrasting biological handling of selenium and tellurium, advancing our understanding of chalcogen biotransformation at the cellular level.

## Supplementary Material

mfag014_Supplemental_File

## Data Availability

The data underlying this article will be shared on reasonable request to the corresponding author.
