# Supplementary material for: Speciation-dependent intracellular reduction and nanostructure formation of selenium and tellurium in unicellular algae
Source: Metallomics. 2026 May 4;18(1):mfag014. doi: 10.1093/mtomcs/mfag014 (PMC13201907; doi:10.1093/mtomcs/mfag014)
Supplement: mfag014_Supplemental_File [file mfag014_supplemental_file.pdf]

Speciation-dependent intracellular reduction and nanostructure formation of selenium and tellurium in unicellular algae

Kohei Odaka [a], Kensuke Inaba [a,b], Yugo Takeuchi [c], Keita Hiraka [b], Daisuke Ogawa [d], Hirokazu Sugimori [d], Kazuhiro Kumagai [e], Akiko Hokura [c*]

a Graduate School of Engineering, Tokyo Denki University, 5 Senju-Asahicho, Adachi, Tokyo 120-8551, Japan
b NISSAN ARC, LTD., 1 Natsushima-cho, Yokosuka, Kanagawa 237-0061, Japan
c Department of Applied Chemistry, 5 Senju-Asahicho, Adachi, Tokyo 120-8551, Japan
d Tokyo Metropolitan Industrial Technology Research Institute, 2-4-10, Aomi, Koto, Tokyo 135-0064, Japan
e Nanodimensional Standards Group, Research Institute for Material and Chemical Measurement National Metrology Institute of Japan (NMIJ), National Institute of Advanced Industrial Science and Technology (AIST), Tsukuba Central 5, 1-1-1 Higashi Tsukuba, Ibaraki 305-8565

*** Corresponding author**

Table S1 EXAFS curve-fitting results for selenium species accumulated in unicellular algae

Algae	Treatment	Exposure time /h	Scattering atom	*N*	*r* /Å	DW /$Å^2$	R-factor /%
C. reinhardtii	Selenate-added	6	C	2.0 *	1.95	0.026	0.67
			O	2.7 *	1.68	0.09	
			Se	2.0 *	2.38	0.12	
		24	C	2.0 *	1.93	0.079	0.31
			O	3.2	2.48	0.14	
			Se	1.8 *	2.36	0.083	
		168	C	2.0	2.09	0.14	0.95
			Se	2.3	2.19	0.12	
	Selenite-added	6	C	2.0 *	2.04	0.037	0.30
			O	3.0 *	1.87	0.14	
			Se	2.2 *	2.39	0.034	
		24	C	2.0 *	2.04	0.035	0.73
			Se	2.0 *	2.38	0.045	
		168	Se	2.0	2.35	0.045	0.17
P. simplex	Selenate-added	6	C	2.0	1.95	0.026	0.67
			O	2.7	1.68	0.090	
			Se	2.0	2.38	0.12	
		24	C	2.0	1.92	0.079	0.31
			O	3.2	2.48	0.14	
			Se	1.8	2.36	0.083	
		168	C	2.0	2.09	0.14	0.95
			Se	2.3	2.19	0.123	
	Selenite-added	6	C	2.0 *	2.08	0.079	0.004
			O	3.0 *	1.73	0.084	
			Se	2.0 *	2.34	0.12	
		24	C	2.0	2.07 *	0.078	0.34
			O	2.9	1.60 *	0.15	
			Se	2.4 *	2.35 *	0.071	
		168	C	2.0 *	2.04	0.085	0.038
			O	3.0 *	1.60 *	0.14	
			Se	2.2 *	2.37	0.063	
Reference materials		Selenate	O	4.0 *	1.66	0.034	0.55
		Reference[a)]		4	1.642	0.0027	0.15
		Selenite	O	3.0	1.71	0.051	1.5
		Reference[a)]		3	1.701	0.0022	0.133
		Se powder	Se	2.7	2.38	0.066	0.30
		Reference[b)]		2.0	2.38	0.0037	0.3
		MeSeCys	C	2.0	1.98	0.027	0.24

Parameters marked with an asterisk were fixed during the fitting procedure.

a) L. Eklund and I. Persson, *Dalton Trans.*, 2014, **43(17)**, 6315-6321. b) W. B. Lu, Z. B. Li, M. J. –Feng, L. R. Zheng, S. C. Liu, B. Yan. J. –S. Hu, D. –J. Xue, *J. Am. Chem. Soc.*, 2024, **146(9)**, 6345-6351.

Table S2 EXAFS curve-fitting results for tellurium species accumulated in unicellular algae

Algae	Treatment	Exposure time /h	Scattering atom	*N*	*r* /Å	DW /Å^2	R-factor /%
C. reinhardtii	Tellurate-added	6h	O	5.0	1.94	0.035	0.85
		24 h	O	4.0 *	1.94	0.036	0.73
		168 h	O	2.5 *	1.95	0.055	0.75
			Te	1.5 *	2.82	0.11	
	Tellurite-added	6h	O	2.5 *	1.90	0.11	2.5
			Te	1.8 *	2.78	0.11	
		24 h	O	2.5 *	1.89	0.13	2.2
			Te	1.7	2.80	0.095	
		168 h	O	2.8 *	1.97	0.10	2.4
			Te	1.8 *	2.76	0.096	
P. simplex	Tellurate-added	6h	O	6.1	1.95	0.019	0.61
		24 h	O	6.3	1.94	0.038	0.32
		168 h	O	6.3	1.98	0.061	0.75
			Te	1.6 *	2.87	0.048	
	Tellurite-added	6h	O	2.8 *	1.90	0.053	0.93
			Te	1.8 *	2.78	0.17	
		24 h	O	2.8	1.91	0.035	5.2
		168 h	O	2.6 *	1.89	0.077	6.0
			Te	1.8 *	2.82	0.11	
Reference materials		Tellurate	O	6.0 *	1.94	0.059	0.62
		Reference[a)]		6.00	1.920	0.004	0.780
		Tellurite	O	2.5	1.88	0.045	0.51
		Reference[a)]		3.00	1.87	0.004	0.006
		Te powder	Te	2.0 *	2.83	0.083	0.14
		Reference[b)]		2.3	2.83	0.006	0.009

Parameters marked with an asterisk were fixed during the fitting procedure.

a) J. L. Goff, Y. W. Wang, M.I. Boyanov, Q. Yu, K. M. Kemner, J. B. Fein and N. Yee, *Environ. Sci. Technol.*, 2021, **55(15)**, 10378−10386. b) T. Kashiwabara, Y. Oishi, A. Sakaguchi, T. Sugiyama, A. Usui, Y. Takahashi, *Geochim. Cosmochim. Acta*, 2014, **131**, 150-163.

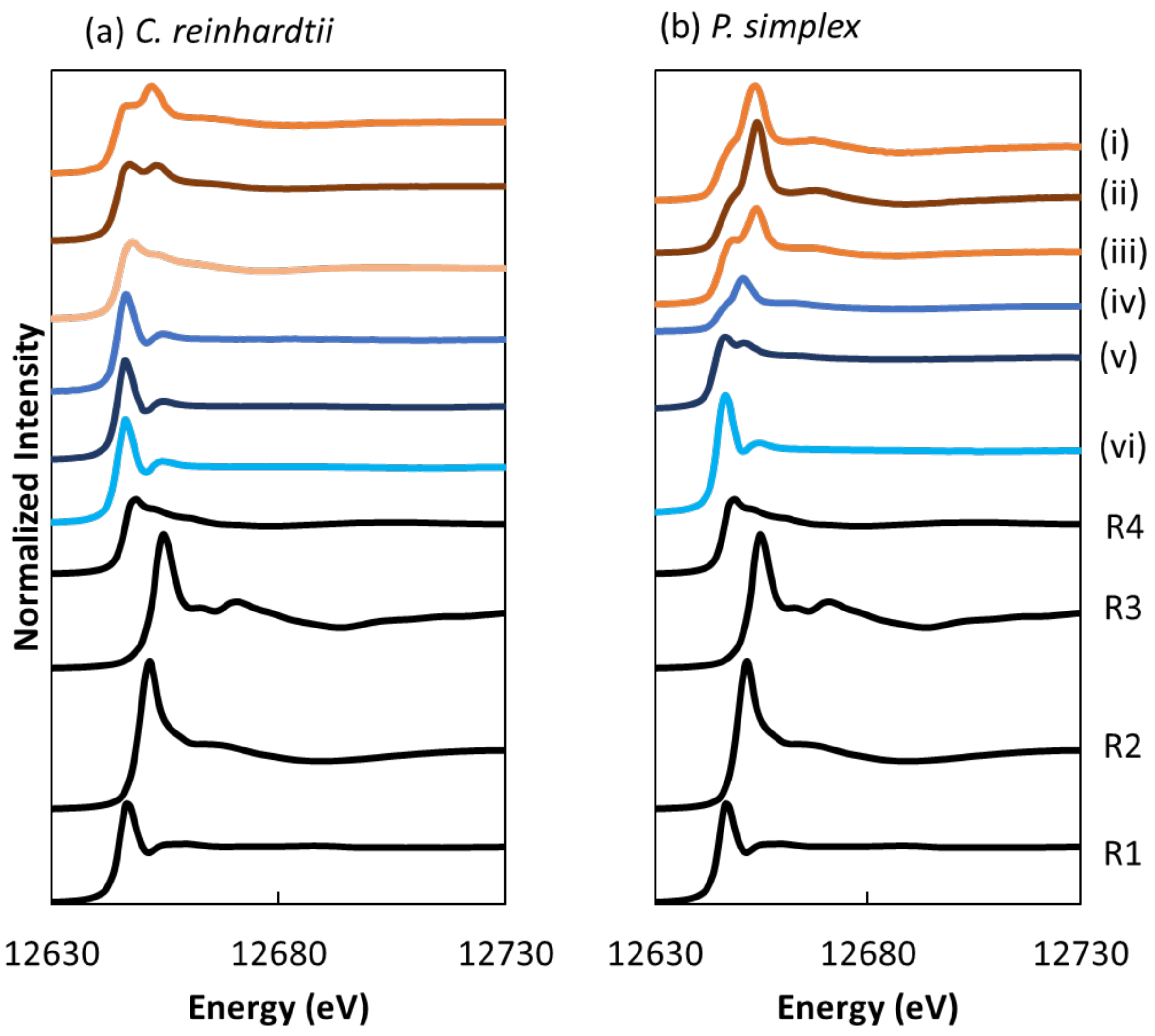

Figure S1 Se K-edge XANES spectra of (a) *Chlamydomonas reinhardtii* and (b) *Pseudococcomyxa simplex* after exposure to selenium oxyanions, together with reference compounds.

Spectra (i), (ii), and (iii) correspond to samples exposed to selenate [Se(VI)] for 6, 24, and 168 h, respectively, whereas spectra (iv), (v), and (vi) correspond to samples exposed to selenite [Se(IV)] for the same incubation times.

Reference spectra are shown for comparison: elemental selenium powder (R1), potassium selenite (R2), sodium selenate (R3), and methylselenocysteine (R4).

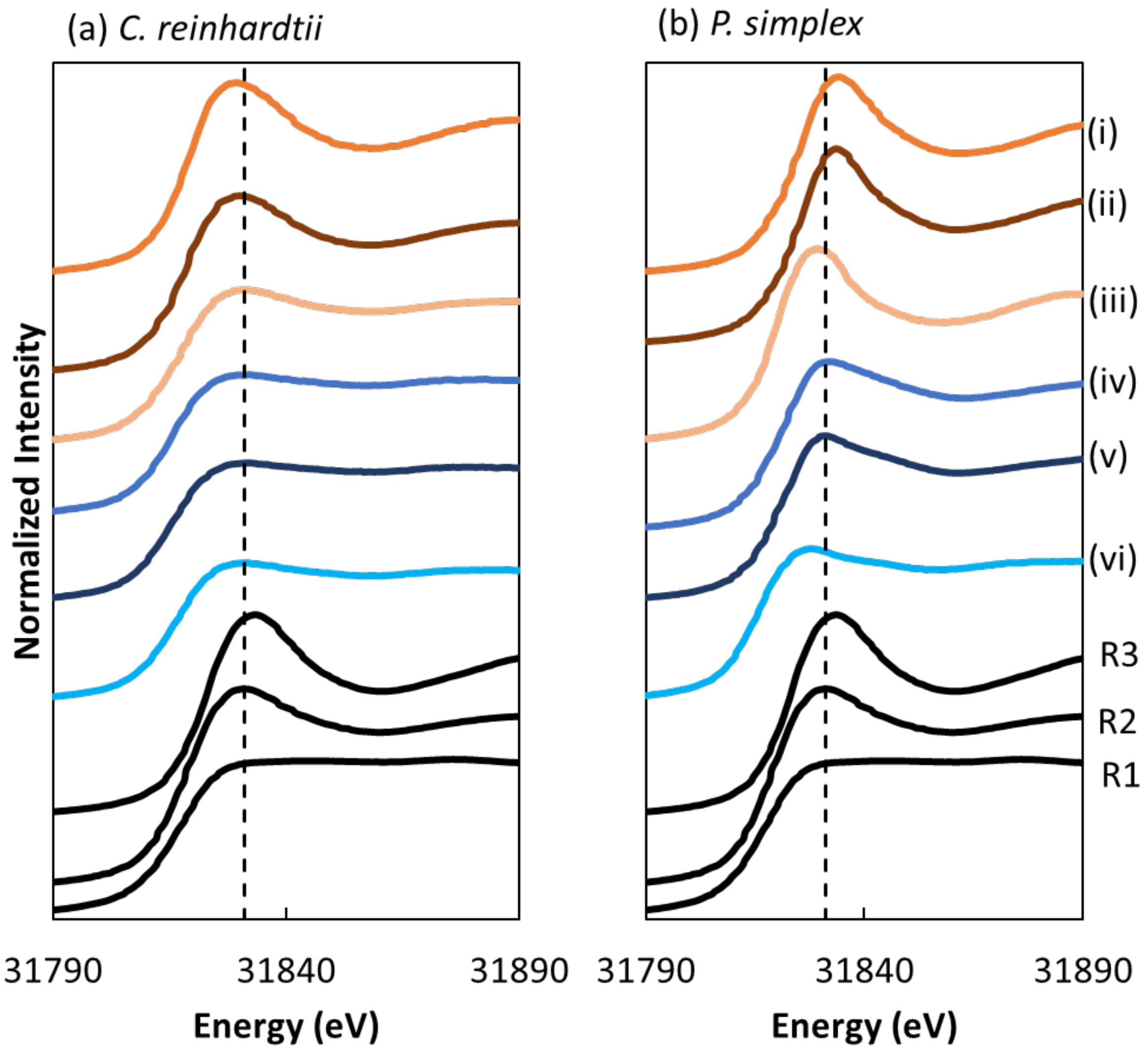

Figure S2 Te K-edge XANES spectra of (a) *Chlamydomonas reinhardtii* and (b) *Pseudococcomyxa simplex* after exposure to tellurium oxoanions, together with reference compounds.

Spectra (i), (ii), and (iii) correspond to samples exposed to tellurate [Te(VI)] for 6, 24, and 168 h, respectively, whereas spectra (iv), (v), and (vi) correspond to samples exposed to tellurite [Te(IV)] for the same incubation times.

Reference spectra are shown for comparison: metallic tellurium powder (R1), potassium tellurite (R2), telluric acid (R3).

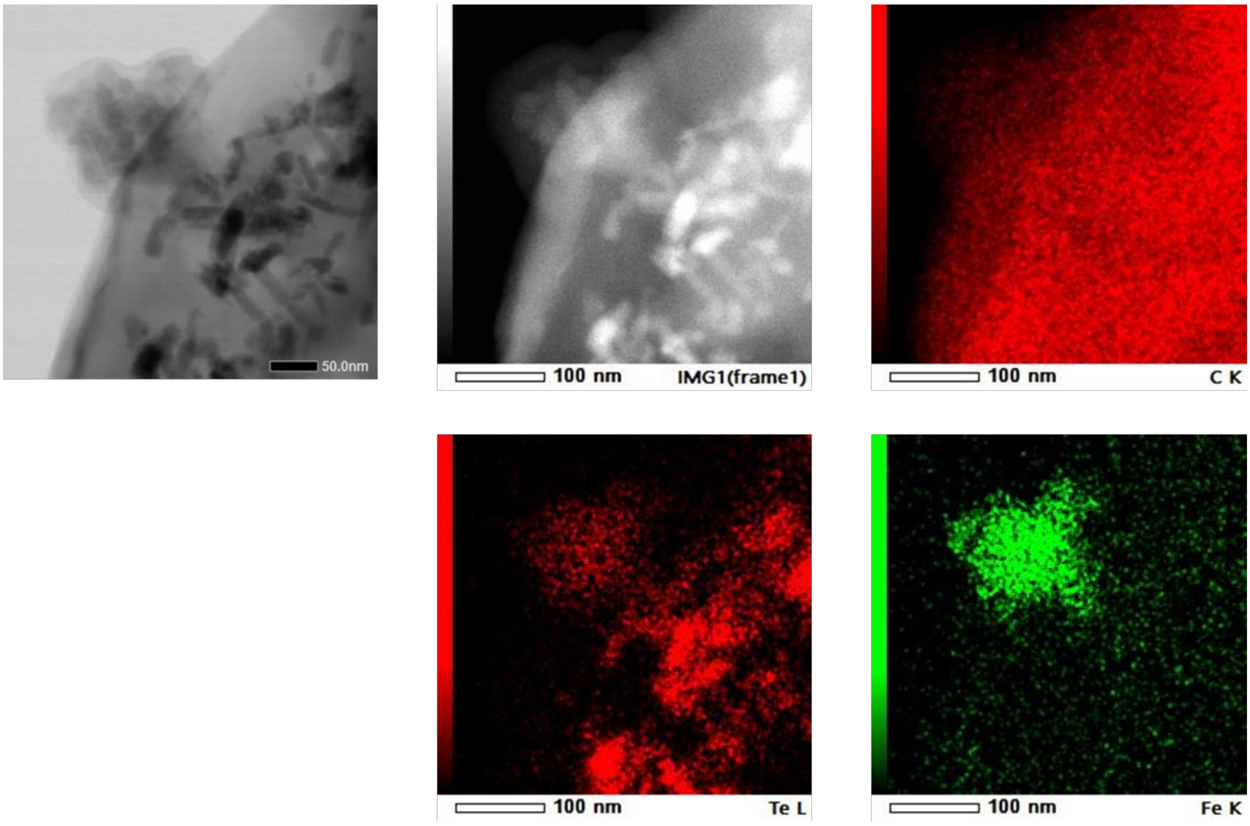

Figure S3　TEM and STEM–EDS data of intracellular tellurium nanorods in *Pseudococcomyxa simplex* after tellurite exposure.

Representative TEM and STEM images together with STEM–EDS elemental maps of *P. simplex* cells exposed to tellurite [Te(IV)] for 7 days (168 h) are shown.

TEM and STEM images show the presence of elongated, needle-like nanostructures within the cell interior.

Elemental mapping reveals that these nanostructures are enriched in tellurium (Te), confirming their assignment as Te nanorods.

The carbon (C) signal reflects the cellular matrix, whereas iron (Fe) is mainly distributed near the cell periphery.

These observations support the intracellular localisation of tellurium nanorods without chemical extraction or sectioning and complement the electron microscopy results presented in the main text.

Scale bars: 50 nm (TEM), 100 nm (STEM and elemental maps).

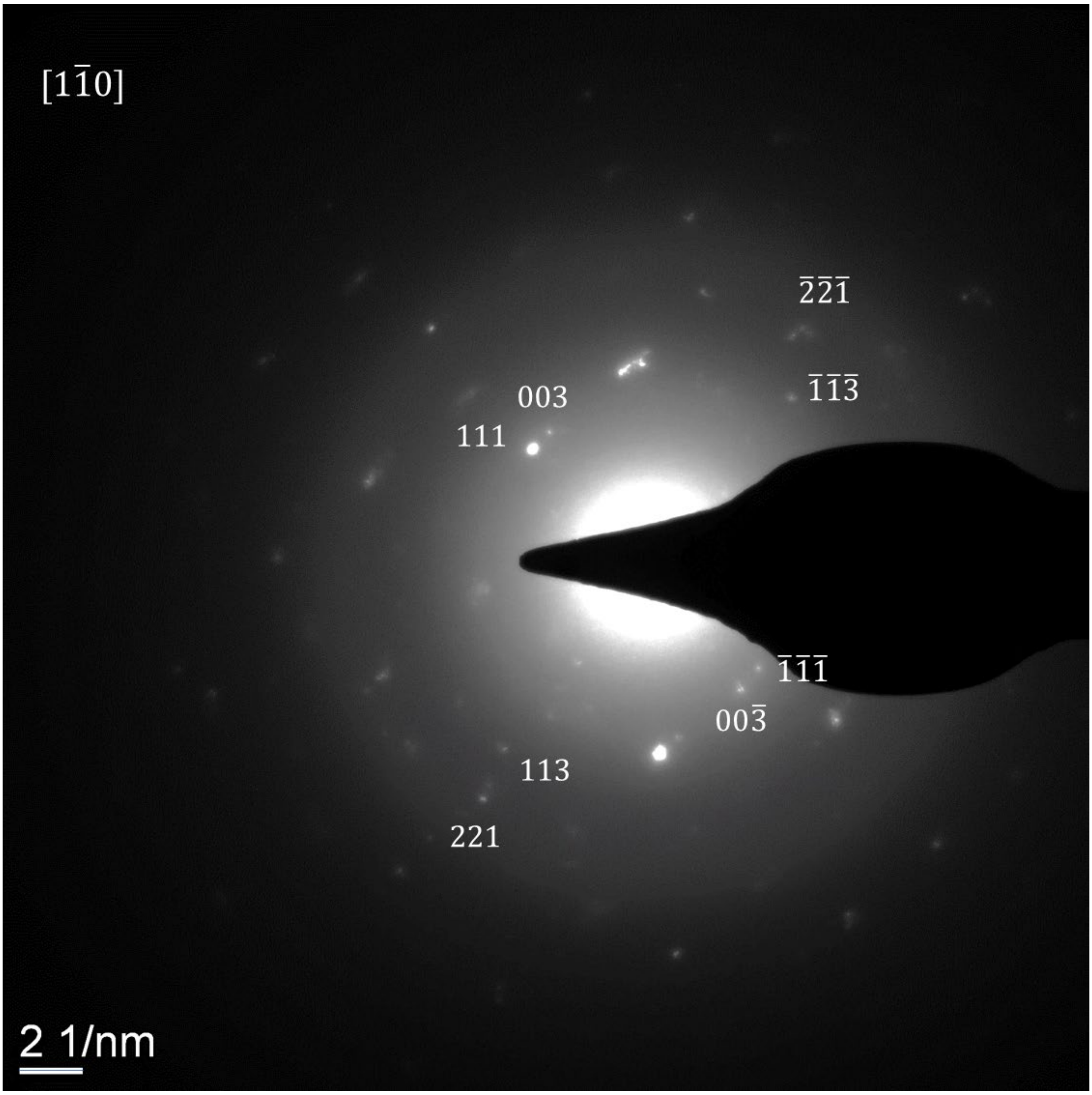

Figure S4 Selected-area electron diffraction (SAED) pattern obtained from intracellular tellurium nanorods in *Pseudococcomyxa simplex*.

The SAED pattern was acquired using an FEI Titan Cubed G2 60-300 transmission electron microscope operated at 300 kV from tellurium nanorods formed inside algal cells after exposure to tellurite [Te(IV)] for 7 days (168 h). The diffraction spots can be indexed to trigonal metallic tellurium, viewed along the [1$\bar{1}$0] zone axis.
